# Intermittent Short Atrioventricular Delay in a Dual Chamber Pacemaker During Acute Pulmonary Edema

**DOI:** 10.1111/anec.70045

**Published:** 2024-12-19

**Authors:** Ravi Vazirani, Miriam García Cocera, David Calvo

**Affiliations:** ^1^ Cardiovascular Institute‐Hospital Clínico San Carlos Madrid Spain

**Keywords:** acute pulmonary edema, AV interval, ECG, Microport, WARAD

## Abstract

We present the case of an 80‐year‐old female with acute pulmonary edema and a dual chamber pacemaker with intermittent short AV delays in the surface ECG after blocked premature atrial contractions (PACs). The behavior was consistent with the programmed Window of Atrial Rate Acceleration Detection (WARAD) and did not require further parameter modifications. As most cardiologists and emergency department physicians are not familiar with brand‐specific algorithms, we believe that this case report will make these noncompetitive atrial pacing algorithms more accessible to non‐cardiologists.

## Graphical abstract

We present the case of an 80‐year‐old female with acute pulmonary edema and a dual chamber pacemaker with intermittent short AV delays in the surface ECG after blocked premature atrial complexes. The behavior was consistent with the programmed Window of Atrial Rate Acceleration Detection and did not require further modifications.

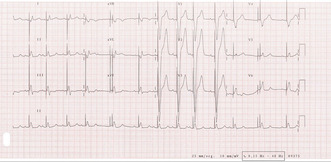

**Abbreviations**
AP: atrial paced
AV: atrioventricular
AVI: AV interval
CCU: cardiac critical care unit
CRT: cardiac resynchronization therapy devices
ECG: electrocardiogram
ELT: endless‐loop tachycardia
ICDs: implantable cardioverter‐defibrillators
PAC: premature atrial contraction
RNRVAS: repetitive nonreentrant ventriculoatrial synchrony
VP: ventricular paced
WARAD: window of atrial rate acceleration detection

1

The cardiology ward was consulted for a hypertensive acute pulmonary edema in an 80‐year‐old woman with a dual‐chamber pacemaker (Microport 2023) implanted for third‐degree atrioventricular (AV) block 3 weeks ago and programmed in DDD(R).

She required intravenous furosemide and nitroglycerin perfusion as well as orotracheal intubation. An electrocardiogram (ECG) was performed on her arrival at the Cardiac Critical Care Unit (CCU) (Figure 1).

**FIGURE 1:**
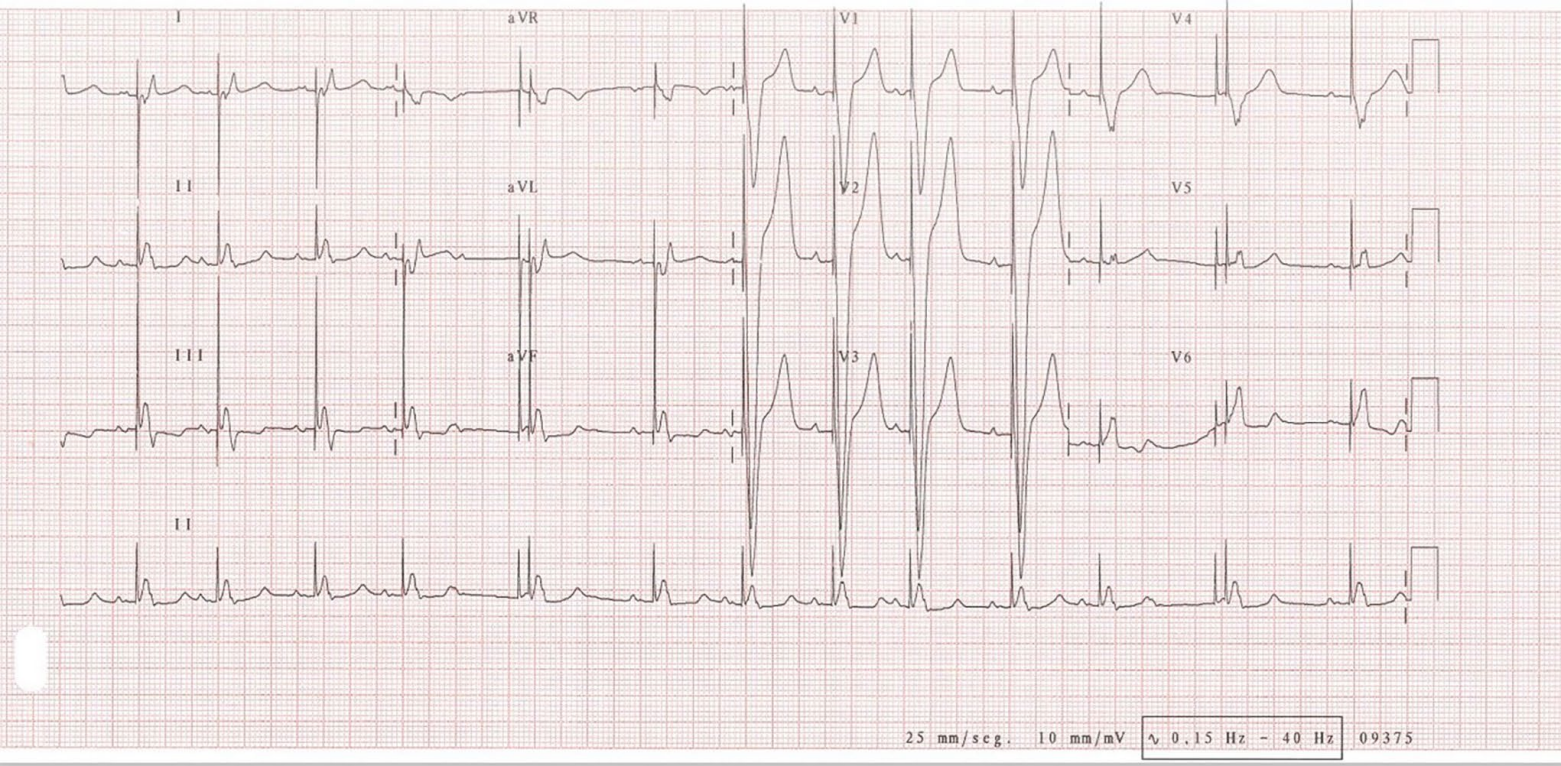
ECG after orotracheal intubation showing sinus rhythm at 78 beats per minute and blocked premature atrial contractions (PACs) triggering an atrial paced–ventricular paced (AP‐VP) event 500 ms after with a short AV interval (fifth beat in the rhythm strip).

The ECG shows a native sinus rhythm, followed by ventricular paced (VP) QRS complexes at a heart rate of 78 beats per minute (bpm). In the fifth beat on the rhythm strip, there is a premature atrial complex (PAC) 400 ms from the last VP that is not conducted to the ventricle, followed by an atrial paced (AP) and VP with a short AV interval (AVi) of 80 ms that occurs 500 ms from the PAC. Sinus rhythm resumes for another six beats until a new PAC triggers a pause and a new AP–VP complex.

### 1.1 Case Report

This is consistent with the Window of Atrial Rate Acceleration Detection (WARAD) developed by Microport (2023); Palacios‐Rubio, González‐Ferrer and Pérez‐Castellano (2019).

### 1.2 Discussion

The WARAD is designed to monitor the atrial activity so as to discriminate pathological atrial waves (eg. atrial fibrillation, atrial flutter or atrial tachycardia) from sinus P waves; it uses atrial prematurity to trigger mode switching. This function is available in all dual chamber Microport (2023) pacemakers, implantable cardioverter‐defibrillators (ICD) and cardiac resynchronization therapy devices (CRT). The WARAD is a dynamic atrial refractory period that is automatically triggered after every atrial event. The duration is automatically calculated by the device and not programmable Microport (2023). If a retrograde P wave falls within the WARAD, the pacemaker does not start an AVi, thus preventing induction of endless‐loop tachycardia (ELT) and repetitive non‐reentrant ventriculoatrial synchrony (RNRVAS) Vazirani‐Ballesteros, Gómez and Fernández‐Jiménez (2023).

When sensing the first PAC in the cycle, the dual chamber device does not initiate an AV delay, and starts:a) a new WARAD of 500 miliseconds max from the PAC Microport (2023); Palacios‐Rubio, González‐Ferrer and Pérez‐Castellano (2019) b) a new atrial escape interval of 500 ms. This behavior is available even if Fallback Mode Switch is OFF and triggers a subsequent AP‐VP complex with short AVi in order to optimize atrial detection. The short AVi is equal to 110 ms, or to the exercise AVi if programmed shorter (as is our case, with 80 ms).

The patient improved from the acute pulmonary edema and was discharged from the CCU 72 h afterwards.

### 1.3 Conclusion

In conclusion, we believe that this case report will make these non‐competitive atrial pacing algorithms more accessible to physicians, who will be aware of them in the future.

## Author Contributions

Conceptualization: R.V. Data curation: R.V. and M.G.C. Formal analysis: R.V. and M.G.C. Investigation: R.V. and M.G.C. Methodology: R.V. Project administration: R.V. Resources: R.V. Software: R.V. Supervision: D.C. Validation: R.V. Visualization: R.V. and M.G.C. Writing: R.V. and M.G.C. Review and Editing: R.V.

## Conflicts of Interest

The authors declare no conflicts of interest.

## Data Availability

The data underlying this article will be shared upon reasonable request from the corresponding author.

## References

[anec70045-bib-0001] Microport. Tech Corner: Window of Atrial Rate Acceleration Detection (WARAD) . 2023. Accessed December 13, 2023. https://microport.com/assets/documents/WARAD_revB_2018.pdf.

[anec70045-bib-0002] Palacios‐Rubio, J. , J. J. González‐Ferrer , and N. Pérez‐Castellano . 2019. “Advanced Pacing Algorithms Resembling Device Malfunction: A Comprehensive Review.” REC: CardioClinics 54, no. 2: 111–126.

[anec70045-bib-0003] Vazirani‐Ballesteros, R. , E. M. Gómez , and R. Fernández‐Jiménez . 2023. “An Under‐Recognized Cause of Pacemaker‐Mediated Rhythm.” European Heart Journal‐Case Reports 7, no. 9: ytad452. 37743901 10.1093/ehjcr/ytad452 PMC10516355

